# Comparison of externally and internally guided dance movement to address mobility, cognition, and psychosocial function in people with Parkinson’s disease and freezing of gait: a case series

**DOI:** 10.3389/fnagi.2024.1372894

**Published:** 2024-05-15

**Authors:** Amit Abraham, Ariel Hart, Ariyana Bozzorg, Suraj Pothineni, Steven L. Wolf, Kersey Schuh, Molly Caughlan, Jelani Parker, Amanda Blackwell, Megan Tharp Cianflona, Courtney Asker, Todd Prusin, Madeleine E. Hackney

**Affiliations:** ^1^Department of Physical Therapy, Faculty of Health Sciences, Ariel University, Ariel, Israel; ^2^Navigation and Accessibility Research Center of Ariel University (NARCA), Ariel University, Ariel, Israel; ^3^Emory University School of Medicine, Department of Medicine, Division of Geriatrics and Gerontology, Atlanta, GA, United States; ^4^University of Georgia, Athens, GA, United States; ^5^Atlanta Veterans Affairs Center for Visual & Neurocognitive Rehabilitation, Decatur, GA, United States; ^6^Emory University School of Medicine Department of Rehabilitation Medicine, Atlanta, GA, United States; ^7^Birmingham/Atlanta VA Geriatric Research Education Clinical Center, Decatur, GA, United States

**Keywords:** Parkinson’s disease, freezing of gait, dance, exercise, internal guidance, cueing

## Abstract

**Objective:**

The aim of this study is to explore the impact of internally guided (IG) versus externally guided (EG) adapted tango (AT) dance training (i.e., dancing the IG “Leader” role or the EG “Follower” role), on motor and non-motor functions in individuals with Parkinson’s disease and freezing of gait (PD-FOG). The “Leader” role, a proxy for IG movements, conveys direction, timing, and amplitude of steps with tactile cues. The “Follower” role, a proxy for EG movements, detects and responds to the leader’s tactile cues.

**Case description:**

Six participants were randomly assigned to the IG (“Leader”) or EG (“Follower”) roles for 20, 90-min AT lessons over 12 weeks. Participants were assessed for PD-specific and non-PD-specific functions before and twice after the end of the 12-week intervention, at 1-week and 1-month post-intervention.

**Results:**

EG participants improved and/or maintained performance on more outcomes across all domains than IG participants. Five participants improved in PD motor symptoms, dynamic gait, global cognitive function, and the FOG Questionnaire immediately or 1 month after intervention. All participants expressed positive attitudes toward the intervention, including improvements in walking, balance, and endurance.

**Conclusion:**

AT training in the follower role may benefit individuals with PD-FOG to a greater extent compared to the leader role.

**Impact:**

This case series study could inform additional research with the goal of enhancing physical therapy or music-based therapy approaches for addressing PD-FOG.

## Introduction

Parkinson’s disease (PD) affects 1% of Americans over 65 (Noyes et al., 2006; Muslimovic et al., 2008; Parkinson’s Foundation, n.d.), impairing quality of life (QOL) (Muslimovic et al., 2008). Freezing of gait (FOG) (Nutt et al., 2011), which is a sudden inability to walk, increases the high fall risk in PD (Balash et al., 2005). Fifty-three percent of people with PD diagnosed for 5 years or more experience FOG (Morris M. E. et al., 2001). People with PD-FOG who participate in physical therapy (PT) exhibit improved gait performance and balance and reduced fall rates (Shen et al., 2016). Such interventions use movement strategies that aim at compensatory neural pathways less affected by PD (Freedland et al., 2002; Abraham et al., 2018). Visual and auditory cueing and treadmill training (Rutz and Benninger, 2020) were effective in addressing PD-FOG (Gilat et al., 2021). Several movement strategies use external guidance (EG) through auditory, visual, or tactile cues (Cunnington et al., 1995; Debaere et al., 2003; Tomlinson et al., 2014). EG training has yielded behavioral benefits in people with PD (Rocha et al., 2014), including movement initiation and reaction times in people with PD-FOG (Dibble et al., 2004; Ballanger et al., 2006; Jiang and Norman, 2006). Audio-visual EG training has improved stride length and velocity in people with PD (Mak et al., 2013). Visual EG strategies have improved movement initiation (Jiang and Norman, 2006) and motor imagery in people with PD (Heremans et al., 2012). Auditory EG strategies can result in faster reaction times in people with PD (Ballanger et al., 2006). Lessened FOG in individuals during turns, which was gained from auditory cues, did not last for a substantially long time (Spildooren et al., 2012). It remains unknown which cue types (auditory, visual, somatosensory, and tactile) or combinations of cued guidance are most effective for improving function in people with PD-FOG.

In contrast, internal guidance (IG), which is a self-initiating movement with no external cues, increases step length and speed of movement in people with PD-FOG (Morris M. E. et al., 2001; Morris et al., 2009; Peterson et al., 2016). People with PD improved their gait more when using IG strategies compared to EG strategies (Harrison et al., 2018). IG strategies may require considerable cognitive engagement (Lau et al., 2004) in executive functions (Elsinger et al., 2006).

In adapted tango (AT) (Allen et al., 2017; Silverstein et al., 2020), participants are assigned to “leader” or “followers” roles. AT is a modified form of Argentine tango, which is a highly improvisational dance. Students learn how to lead steps through subtle pressure cues through the arms in the “frame.” Students in AT are encouraged to improvise the order of steps in their dances. Partners are trained to coordinate their steps by paying close attention to the tactile cues, and the indicated directions through the body’s posture. The leader plans and executes the timing, direction, rotation, and amplitude of the steps, requiring IG of their movements. As such, in this study, we consider the lead role to be mostly but not exclusively an IG strategy. The follower uses EG to make the next move by responding to the leader’s tactile and visual guidance and cues about what the next step will be and how it will be executed. Therefore, in this study, we consider the follower role to mostly be using an EG strategy as pertains to what the next dance steps will be. We acknowledge that both of these roles, which involve human movement, require I and EG of movement; however, for the follower to know where to go next because the dance is improvisational, the follower must pay attention to the leader’s cues, which provide information about where, how far, and when to step next. The leader, on the other hand, is not being told what the next step will be and therefore must self-determine the next steps and all the parameters about that step. For people with PD, research has shown that AT training incorporates visual, auditory, and, most importantly, tactile cueing to improve motor deficits, disease severity, and cognition in people with PD (Hackney and Earhart, 2010).

Previous studies of AT in PD did not examine the effect of AT on FOG; however, the studies included participants both with and without FOG without taking this difference into consideration (Hackney et al., 2007; Hackney and Earhart, 2009, 2010; McKee and Hackney, 2013). The goal of this exploratory case series is to evaluate the effects of IG-AT training versus EG-AT training on motor, cognitive, and psychosocial functions in six individuals with PD-FOG. As people with PD and FOG exhibit impairments in planning and executing complex goal-directed tasks (Kliegel et al., 2005), we hypothesize that people with PD-FOG will exhibit greater benefits from EG-focused (i.e., follower) AT training, compared to IG-focused (i.e., leader) AT training.

## Narrative of the episode of care

The Institutional Review Board at Emory University School of Medicine and the Department of Veterans Affairs Rehabilitation Research & Development committee approved the study. This report adheres to the 2013 CARE case report guidelines (Writing a Case Report, n.d.). Participants provided written informed consent before participation. Participants had idiopathic definite PD (Racette et al., 1999) no other neurological insult, and clinically significant FOG, defined as reporting freezing at least “once a week” on item 3 of the Freezing of Gait Questionnaire (FOGQ): “Do you feel that your feet get glued to the floor while walking, making a turn or when trying to initiate walking (freezing)?” (Giladi et al., 2001; Moore et al., 2007), exclusion criteria were: major psychiatric illness, history of stroke or traumatic brain injury, alcohol abuse and/or use of antipsychotics, severe cardiac disease, and other significant co-morbid diseases. Participants underwent three evaluations while “off” medications. One week before (pre-test) and after finalizing the intervention: directly 1 week after (post-test), and 4–6 weeks after (1-month post). At the post-test, participants completed an exit questionnaire to assess satisfaction and whether they noted improved balance, walking, mood, coordination, strength, and endurance after the intervention (Hackney et al., 2013; McKee and Hackney, 2013). Participants were randomly assigned to the IG (leader) or EG (follower) roles before baseline assessment. Participants received 20, 90-min biweekly AT classes within 12 weeks. Trained, blinded raters administered assessments.

## Participants’ demographics and clinical characteristics

Participants were Caucasian (*n* = 4), Black (*n* = 1), and Asian/Caucasian (*n* = 1) and were in Hoehn and Yahr stages 2.5–4, had PD 8–18 years, and reported moderate to high fear of falling. All participants were being followed by a movement disorders specialist, reported fully adhering to their medical regimen, and benefited from anti-parkinsonian medication. All but one participant (EG2) reported the right onset of Parkinson’s. Five of the six were considered “fallers,” having reported more than two falls in the previous 6 months, with EG3 being a daily faller (182 falls) and IG1 reporting no falls. Participants were all retired, lived independently, and were aged 61–78 years. Participants ranged in exercise habits from daily (IG1) to 1–2 days/week (IG3, EG1, and EG3) to occasional walking only (IG2 and EG2). Four participants used an assistive device, and one (EG2) reported occasional use of a wheelchair. Participants reported moderately good to high QOL on the single-item QOL measure (Table 1).

**Table 1:** Participants’ demographics and medical history.

	IG1	IG2	IG3	EG1	EG2	EG3
Group	Internally guided training	Internally guided training	Internally guided training	Externally guided training	Externally guided training	Externally guided training
Age (y)	66	71	69	68	78	61
Sex	Male	Female	Male	Male	Female	Female
Race	Caucasian	Black	Caucasian	Caucasian	Caucasian	Asian/Caucasian
Time since diagnosis (y)	8	12	16	16	14	18
Hoehn & Yahr stage	2.5	3	2.5	3	4	3
Disease onset	Right	Right	Right	Right	Left	Right
No. of falls in 6 months before the study	0	3	3	6	7	182
Observed FOG during MDS-UPDRS assessment	No	Yes	No	Yes	Yes	Yes
Freezing of gait questionnaire (/24 total score) (item 3)	12 (3)-(about once a day)	6 (2)-(about once a week)	14 (2)-(about once a week)	12 (3)-(about once a day)	4 (4)-(whenever walking)	20 (3)-(about once a day)
Composite physical function (CPF) index (/24)	Moderate functioning (20)	Moderate functioning (16)	High functioning (24)	High functioning (24)	Low functioning (7)	Moderate functioning (18)
Fear of falling (/7)^^^	7	3	4	4	5	7
Quality of life (/7)^^^	4 (moderate)	5 (moderately good)	5 (moderately good)	3 (low)	4 (moderate)	6 (high)
Beck depression inventory II	20	9	12	12	23	23
Physical activity scale for the elderly (PASE)	62.01	130	190.7	145.1	75.53	53.6
ABC scale (/100)	66.9	86.3	68.1	91.3	68.1	50.6
Medications	PD specific: Carbidopa-Levodopa 25 mg-100 mg tab; Non-PD Specific: Bupropion HCL XL 150 mg, Myrbetriq ER 50 mg, Omeprazole DR 40 mg, Cialis 5 mg	PD specific: Carbidopa-Levodopa 25 mg-250 mg, Selegiline 5 mg; Non-PD Specific: Seroquel 25 mg, Carvedilol 12.5 mg, Nexium, Levothyroxine 0.125 mg, Magnesium citrate, Meclizine 25 mg, Pravastatin 20 mg, Travatan Z, Ubiquinone 100 mg, Diovan 160 mg	PD specific: Carbidopa-Levodopa 25 mg-100 mg, Mirapex 0.5 mg, Amantadine 100 mg, Comtan 100 mg; Non-PD Specific: Trazadone 100 mg, Coumadin/Warfarin 2 mg, Simvastatin 40 mg	PD specific: Carbidopa-Levodopa 25 mg-100 mg; Non-PD Specific: Bupropion HCL XL 150 mg, Omeprazole DR 4omg, Myrbetriq ER 50 mg, Cialis 5 mg	PD specific: Carbidopa-Levodopa 25 mg-200 mg, Amantadine 100 mg; Non-PD Specific: Lexapro 10 mg, Lortab 5/500 mg, Atorvastatin 20 mg, Cyanocobalamin 100mcg/mL, Colace 100 mg, Nexium 40 mg, Lunesta 2 mg, Proctocort 30 mg, Lidocaine 5% topical ointment, Metoprolol tartrate 25 mg, Conjugated estrogens 0.625 mg/g, Premarin 0.45 mg	PD specific: Carbidopa-Levodopa, Mirapex, Entacapone, Amantadine; Non-PD Specific: Mirtazapine

^*^Freezing of gait questionnaire (FOGQ) Item 3 (/4): “Do you feel that your feet get glued to the floor while walking, making a turn or when trying to initiate walking (freezing)?”^^^Fear of falling and quality of life are self-rated on a 7-point Likert scale, from 0 to 7, with higher scores indicating more fear of falling or quality of life.MDS-UPDRS: movement disorders society unified parkinson disease rating scale.

### Outcome measures

#### PD-specific outcome measures

FOGQ (subjectively evaluates FOG frequency and disturbances in gait, unrelated to falls) (Freezing of Gait Questionnaire, n.d.), the Movement Disorders Society Unified Parkinson Disease Rating Scale revision (MDS-UPDRS) parts I-IV (Movement Disorder Society—Sponsored Unifed Parkinson’s Disease Rating Scale Division, 2014), and the Hoehn and Yahr (1967) and Goetz et al. (2004) scale, and the Parkinson Disease Questionnaire-39 (PDQ-39; summary index score and the ADLs subscale score) (Parkinson’s Disease Questionnaire-39, 2014) were used to measure the PD-specific outcomes.

#### Mobility and fall risk outcome measures

The Timed Up and Go [TUG; shorter times indicate a lower risk of falls (Vance et al., 2015)] (Morris S. et al., 2001), Manual Timed Up and Go (TUG-M) (Shumway-Cook et al., 2000), Cognitive Timed Up and Go (TUG-C) (Campbell et al., 2003), Dynamic Gait Index (DGI) (Landers et al., 2008; Huang et al., 2011), Gait Speed (Steffen and Seney, 2008; Fritz and Lusardi, 2009; Abu Samah et al., 2016), Four-Square Step Test (FSST) (Duncan and Earhart, 2013), the Fullerton Advanced Balanced Scale [FAB; measures static and dynamic balance in older active adults and those with PD (Schlenstedt et al., 2015)] (Fullerton Advanced Balance Scale, 2012; Physical Activity Scale for the Elderly (PASE), 2016), and composite physical function (CPF) index (Fullerton Advanced Balance Scale, 2012) were used to measure the mobility and fall risk outcomes.

#### Psychosocial outcome measures

The Activities-Specific Balance Confidence (ABC) scale (score < 69%indicates risk of recurrent falls. Fear of Falling and QOL are self-rated on a 0–7 Likert scale, with “7” indicating greater fear of falling and better QOL, respectively) (Activities-Specific Balance Confidence Scale, 2013), Short Form-12 Health Survey (SF-12; participant’s perspective of health-related QOL with composite scores for physical and mental health. Higher scores on the SF-12 are better) (Short Form 12 Item (version 2) Health Survey, n.d.), and Beck Depression Inventory II (BDI-II; assesses depression, with score > 18 indicating depression in PD) (Beck Depression Inventory, 2012) were used to measure the psychosocial outcomes.

#### Cognitive outcome measures

The following cognitive outcome measures were administered: Trail Making Test (Reitan, 1955) (for visual attention and task switching), Brooks Spatial Memory (BSM) (Brooks, 1967) (for spatial memory), The Delis-Kaplan Executive Function System (D-KEFS) Tower of London Test (for planning and problem solving), The D-KEFS Color Word Interference Test (CWIT) (Delis et al., 2001) (for executive function over four conditions: color naming, word reading, inhibition and inhibition/switching) (Long et al., 2015), the Montreal Cognitive Assessment (MoCA) (2012), the Reverse Corsi Blocks (Kessels et al., 2008) test (for visuospatial working memory), and the Serial 3’s task (Bristow et al., 2016) (for mental tracking capacity and updating). MoCA cut-off scores were as follows: >26/30 indicates screened for normal cognitive function, <26/30 indicates possible mild cognitive impairment, and <18/30 indicates possible PD-dementia.

The Reverse Corsi Blocks (Kessels et al., 2008) test (for visuospatial working memory) and Serial 3’s task (Bristow et al., 2016) (for mental tracking capacity and updating).

### Baseline assessment

Participants’ baseline performance on PD-specific, mobility, and cognitive outcomes is presented in Tables 1, 2.

**Table 2:** Outcome performance^a^.

	EG1			EG2			IG1			IG2			IG3			EG3		
	Pre	Post	1MTH post	Pre	Post	1MTH post	Pre	Post	1MTH post	Pre	Post	1MTH post	Pre	Post	1MTH post	Pre	Post	1MTH post
Movement disorders society unified parkinson’s disease rating scale																		
I. Non-motor experiences of daily living (/52)	22	20	16	16	7	8	29	21	16	14	18	16	13	11	7	14	13	14
II. Motor experiences of daily living (/52)	19	18	15	32	18	26	31	24	28	13	11	13	14	18	19	17	19	17
III. Motor examination (/132)	48	39	34	57	57	55	56	47	44	40	32	31	31	31	29	46	49	51
IV. Motor complications (/24)	6	4	6	1	3	0	6	1	9	0	3	6	10	8	12	9	9	6
MDS-UPDRS total (/260)	95	81	71	106	85	89	122	93	97	67	64	66	68	68	67	86	90	88
Freezing of gait questionnaire (/24)	12	13	12	17	22	18	12	13	10	6	6	6	14	14	13	20	16	17
Gait speed fast	1.45	1.51	1.49	0.55	0.30	0.69	1.52	1.61	1.40	1.12	1.13	1.27	1.22	1.11	1.14	1.53	1.65	1.69
Gait forward	1.11	0.87	1.08	0.38	0.21	0.53	0.86	1.00	0.76	0.76	1.14	0.98	0.89	0.73	1.05	1.09	1.18	1.07
Backward	0.74	0.77	0.79	0.13	0.06	0.09	0.57	0.46	0.56	0.29	0.26	0.41	0.79	0.64	0.74	0.40	0.42	0.56
Timed up and go (TUG) (s)	7.7	9.4	8.5	50.0	95.5	22.4	8.8	12.3	9.2	11.4	11.71	11.0	10.4	9.9	10.3	9.0	9.7	8.7
TUG-cognitive (s)	9.3	10.8	10.4	167.1	206.9	72.5	17.7	28.4	16.1	16.6	14.6	15.0	11.8	20.1	14.3	14.3	20.6	14.8
TUG-manual (s)	10.0	14.6	13.4	133.1	–^c^	91.9	17.4	12.8	17.4	14.7	12.8	13.3	12.4	14.0	17.4	11.6	13.6	12.7
Dynamic gait index (/24)	16.0	18.0	22.0	2.0	11.0	16.0	19.0	21.0	21.0	18.0	20.0	21.0	23.0	19.0	19.0	18.0	18.0	19.0
Fullerton advanced balance (/40)	19.0	29.0	31.0	13.0	6.0	16.0	24.0	25.0	19.0	23.0	22.0	20.0	35.0	35.0	36.0	20.0	26.0	23.0
Four-square step test (s)^b^	8.8	9.0	8.2	24.2	31.3	–^c^	14.2	13.9	13.9	15.6	14.5	14.2	13.3	10.3	9.8	9.0	10.2	14.2
Composite physical function (/24)	24.0	24.0	23.0	7.0	7.0	9.0	20.0	21.0	16.0	16.0	14.0	16.0	24.0	18.0	22.0	18.0	17.0	17.0
Parkinson’s disease questionnaire (PDQ-39)																		
Summary index	33.6	13.7	21.6	60.4	26.3	27.8	47.8	49.0	38.3	10.5	19.3	34.3	30.3	25.9	26.4	37.4	55.6	35.9
PDQ-39 ADL	25.0	20.8	12.5	75.0	33.3	16.7	50.0	41.7	41.7	4.2	4.2	4.2	37.5	29.2	25.0	37.5	66.7	33.3
PDQ-39 stigma	31.3	0.0	6.3	75.0	0.0	0.0	25.0	0.0	12.5	12.5	6.3	43.8	12.5	6.3	0.0	0.0	37.5	18.8
PDQ39 mobility	15.0	7.5	0.0	75.0	85.0	47.5	42.5	52.5	25.0	17.5	12.5	35.0	55.0	30.0	30.0	47.5	95.0	25.0
PDQ-39 emotional well-being	37.5	12.5	41.7	75.0	33.3	62.5	37.5	41.7	20.8	0.0	0.0	0.0	20.8	4.2	20.8	16.7	41.7	25.0
PDQ-39 social support	41.7	8.3	25.0	91.7	16.7	41.7	16.7	50.0	16.7	0.0	0.0	50.0	25.0	8.3	37.5	0.0	8.3	8.3
PDQ-39 cognition	43.8	18.8	37.5	0.0	0.0	12.5	68.8	56.3	56.3	0.0	31.3	50.0	25.0	37.5	31.3	81.3	62.5	68.8
PDQ-39 bodily discomfort	33.3	41.7	25.0	58.3	41.7	25.0	91.7	75.0	66.7	25.0	50.0	41.7	41.7	58.3	41.7	41.7	33.3	58.3
PDQ-39 communication	41.7	8.3	25.0	33.3	0.0	16.7	50.0	75.0	66.7	25.0	50.0	50.0	25.0	33.3	25.0	75.0	100.0	50.0
Beck depression inventory	12	17	15	23	8	13	20	11	16	9	10	0	12	12	9	23	20	18
SF12 physical composite score	47.3	49.9	55.0	31.2	37.6	51.7	37.9	40.9	36.8	47.1	36.7	41.6	28.7	39.6	46.6	47.8	46.4	42.0
SF 12 mental composite score	40.9	44.0	41.9	35.6	44.4	42.7	31.6	34.2	42.9	50.2	46.7	44.2	51.2	48.5	39.3	31.5	26.2	27.3
Activities-specific balance scale (%)	91.3	87.5	83.8	68.1	75.6	70.6	66.9	76.3	78.8	86.3	80.6	89.4	68.1	63.8	66.3	50.6	47.5	21.9
Montreal cognitive Assessment (/30)	25	28	28	21	26	22	19	24	21	24	22	26	24	25	26	28	29	28
Tower of london achievement score	15	18	15	11	9	14	4	0	2	8	6	10	10	13	16	17	19	20
Trails making test-A	25.1	23.1	27.1	59.2	45.9	67.6	88.3	142.8	136.3	65.8	35.3	26.4	23.7	31.5	27.9	28.4	25.4	20.8
Trails making test-B	70.4	63.8	79.0	300^d^	182.8	185.4	300^d^	300^d^	300^d^	159.2	190.6	147.7	140.1	96.1	150.4	44.3	47.0	74.6
Trails difference (B-A)*	45.3	40.7	51.9	N/A	136.9	117.8	N/A	N/A	N/A	93.4	155.3	121.3	116.4	64.5	122.5	16.0	21.6	53.9
CWIT-d inhibition completion time (s)	9	7	11	5	9	2	2	1	–^c^	2	7	7	4	2	6	8	11	11
CWIT-d Inhibition/switching Completion time (s)	11	13	13	9	10	9	2	–^c^	–^c^	6	5	7	8	1	8	10	13	13
CWIT-d inhibition total corrected and uncorrected errors	12	8	12	9	13	10	1	1	–^c^	11	11	13	8	9	10	1	8	4
CWIT-d inhibition/switching total corrected and uncorrected errors	4	12	11	6	9	4	1	–^c^	–^c^	7	6	12	8	5	7	9	9	10
Serial 3’s (% correct)	100.0	97.8	90.5	100.0	94.1	100.0	75.0	68.4	85.0	84.2	100.0	100.0	100.0	100.0	100.0	93.8	96.0	85.7
Corsi blocks product (span * number correct trials)	20	42	20	24	30	20	9	6	16	20	30	30	42	20	20	40	40	48
Brooks spatial memory (% correct)	60	72	–^c^	54	58	70	36	24	20	64	44	52	64	68	86	62	58	58

^a^Participants are sorted from left to right according to progressively worse performance. Green cells represent improved or maintained performance compared to pre-test. Red cells indicate declined (i.e., more than 5%) performance relative to pre-test.^b^Scores scaled to normative data. CWIT = color-word interference test.^c^Participant was unable to complete the test.^d^Participant exceeded the time allowed to complete the test and did not complete the test.

#### PD-specific

IG1 and IG3 did not exhibit FOG during MDS-UPDRS-III item 11 (FOG). Other participants demonstrated mild to moderately severe FOG during the MDS-UPDRS assessment. On MDS-UPDRS Parts I and II, all participants reported moderate to severe difficulty. On MDS-UPDRS Part III, all participants had moderate severity. All participants—besides IG2—reported medication-related motor fluctuations per the MDS-UPDRS IV. On PDQ-39, all participants—except IG2, who scored below the mean—had normative scores for health-related QOL (Parkinson’s Disease Questionnaire-39, 2014).

#### Mobility and fall risk

Scores on DGI, which were less than 19/24, and FAB, which were less than 25/40, indicated elevated fall risk in all participants, except EG3. A score of <19/24 on the DGI is predictive of falls, while scores of 22 or above indicate safe ambulators (Landers et al., 2008; Huang et al., 2011). Lower FAB scores indicate difficulty with higher level static and dynamic balance tasks. A score of <25/40 is predictive of increased fall risk (Schlenstedt et al., 2015).

The FSST results for all participants—except EG1 and EG3—indicated elevated fall risk because a score of more than 9.7 s to complete the FSST is predictive of increased fall risk. Participants’ retropulsion scores on the MDS-UPDRS-III and their Hoehn and Yahr ratings indicate postural instability because a score of 2.5 or greater on the Hoehn and Yahr, which all participants had, is indicative of the cardinal sign of postural instability (determined by the retropulsion test, in which patients took three or more steps to recover, or were caught by the examiner). On the CPF, only EG1 and IG3 were considered “high functioning” in performing ADLs without help; other participants were not fully independent with all tasks. EG2 was a “low functioning” participant and the most at risk for declining physical independence. On the CPF, a score of ≥14 indicates moderate functioning, and a score of <14 indicates low functioning.

#### Psychosocial

Participants IG1, IG3, EG2, and EG3 had low balance confidence, per ABC scores. On BDI-II, IG1, EG2, and EG3 scores indicated significant depression. All participants fell under the mean score of 50 on the physical and mental composite score for the SF-12, indicating worse self-reported physical and mental health. On PASE, IG1 and EG3 reported lower levels of physical activity (Washburn et al., 1993).

#### Cognitive

On MoCA, at baseline, all participants (range MOCA: 21–25) but EG3 (MOCA score: 28) exhibited possible mild cognitive impairment (score < 26/30) (Chou et al., 2010). On Corsi blocks, IG1, IG2, EG1, and EG2 scored significantly lower than their age group’s normative score, whereas IG3 and EG3 scored higher (McKee and Hackney, 2013; Abraham et al., 2018).

### Therapeutic interventions

Participants attended 20, 90-min AT classes at the Atlanta VA Medical Center over 12 weeks and were encouraged to attend two classes per week. All classes included review/practice, warm-up, rhythmic entrainment/partnering enhancement, new steps, combining previous steps, and cool down (Hackney and McKee, 2014) (Table 3). People with PD always danced with individuals without PD (i.e., caregivers or student volunteers).

**Table 3:** Adapted tango class sections and implications for participants’ roles.

Class section/time	Implications for IG and EG participants
Greeting and review: practice/10 min	Both groups practice steps from the previous classes.
	IG participants create movements step sequences and initiate them during the warm-up, allowing them to remember previously learned moves and be more creative for their partners. After 10 lessons, they will have a larger bank of moves to pull from, increasing the difficulty of selecting moves to lead their partner through.
	EG participants follow tactile cues via tactile cues on their forearms and hands from their partner and do not determine timing, rotation, direction, or amplitude of steps.
Warm-up/20 min	Instructor leads exercises to emphasize range of motion in joints from head to toe, rotational movement of the trunk, contra-body motion, and focus on breathing and postural alignment.
Rhythmic entrainment/partnering enhancement/20 min	Enhances the participant’s feel of the musical beat.
	IG participants are instructed on how to effectively communicate via tactile forces and pressure movement goals (e.g., amplitude, timing, and rotation) to the EG participants.
	EG participants increase their ability to respond to the tactile cues from the IG participants.
New step/15 min	A new step is taught in every class.
	IG participants lead EG partners through the new step, using tactile cues via force and pressure through the hands and arms of the dance frame position.
	EG participants respond to the tactile cues from the IG participant.
Combining previous steps/20 min	Participants are given three steps to practice, allowing for increased repetition and creativity in combining the steps.
	IG participants are responsible for deciding how to combine the three given practice steps (in a self-determined order, and number of iterations per step), allowing for increased repetition and creativity in combining the steps.
	EG participants follow the IG participants through the three practice steps for the session, responding only to tactile cues. Ideally, after 10 lessons the EG participant has an enhanced ability to strictly follow cues from the IG participant, refraining from anticipating/initiating movements without proper cueing.
Closing/cool down/5 min	Gather in a circle to finish with breathing exercises and stretching techniques, bringing the heart rate back down. Discuss class accomplishments and build a community learning environment.

### Follow-up and outcomes

All participants completed 20 lessons within 12 weeks. IG2 and EG3 experienced near-falls during AT sessions, which were prevented by AT partners. EG3 experienced a non-injurious fall during a FOG episode while turning, resulting in a loss of balance and an eventual fall to the ground. She was immediately attended to by a trained clinician, and she could continue therapy.

**Figure 1:**
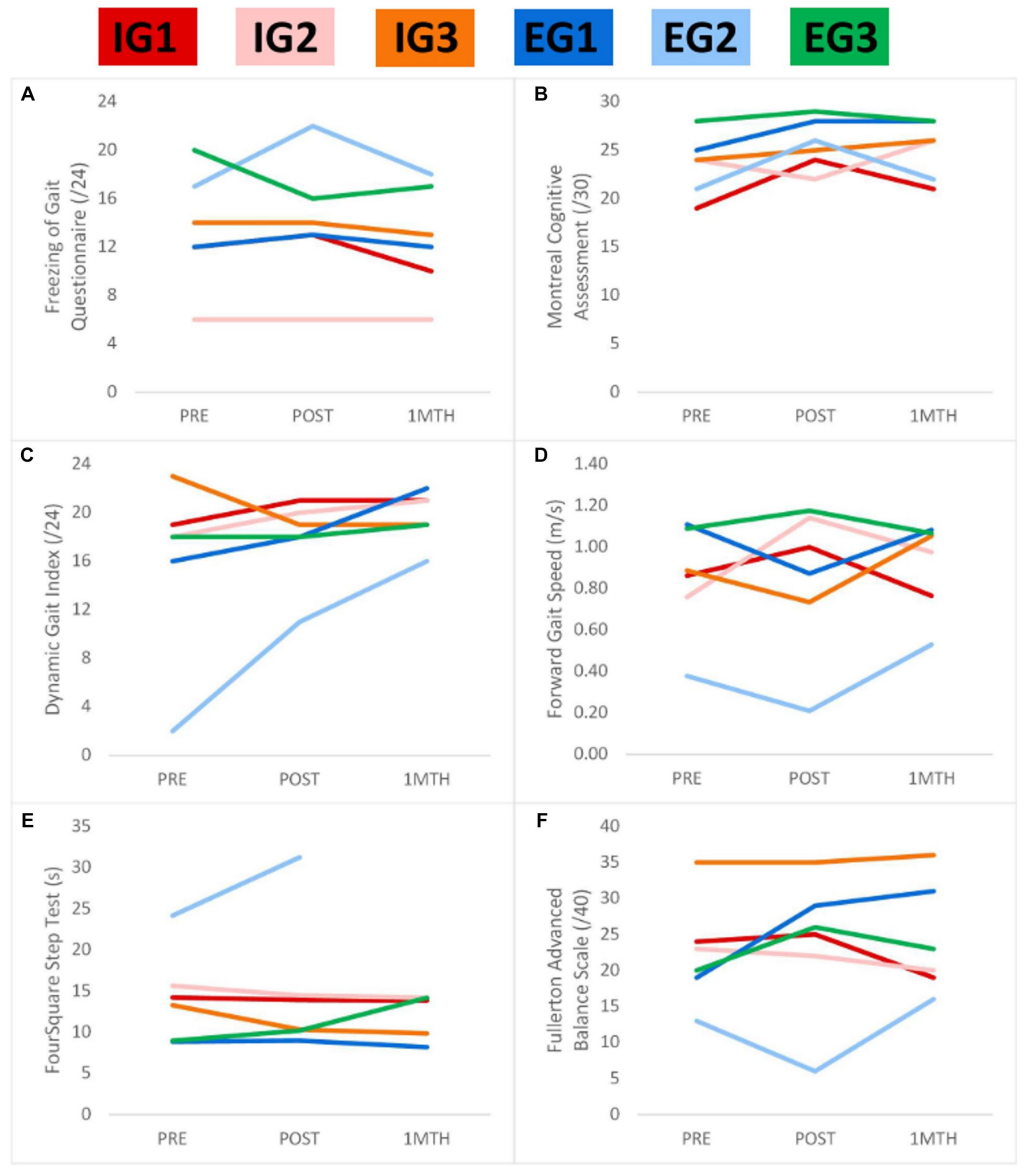
Performance over the trial on the freezing of gait questionnaire **(A)**, montreal cognitive assessment **(B)**, dynamic gait index **(C)**, forward gait speed **(D)**, four-square step test **(E)**, and fullerton advanced balance scale CEL legend at the top shows the colors of participants. Self-reported freezing of gait **(A)** was maintained for most participants. Most participants improved in global cognition **(B)**, gait **(C,D)**, balance **(C,F)**, and mobility/motor cognition **(E)**. One-month post-test data were not available for EG2 for FSST. *Lower scores for FSST and FOGQ indicate better outcomes.

After intervention, EG-AT improved or maintained performance more consistently on 23 outcome variables, while IG-AT improved/maintained performance more consistently on 12 outcomes, and both IG-AT and EG-AT improved/maintained performance equally on 6 outcomes (meaning that there were more timepoints with improvement or maintenance across all three participants in a group compared to that of the other group) (Table 2). Five participants improved global cognition and reported less detriment to QOL. All participants, except EG2, improved FOGQ scores, either at post-test or 1-month post-test. EG3, who had severe freezing, improved both at the post-test and 1-month post-test. Participants improved in outcome measures across all domains, regardless of whether they were assigned to the EG-AT or IG-AT roles (Table 2). All participants besides IG3 improved dynamic balance, per the DGI. All IG-AT participants and EG1 improved on FSST. DGI scores were maintained or improved at the post-test and further improved at the 1-month post-test for all participants except IG3. MoCA and MDS-UPDRS total scores showed consistent responses over time, regardless of EG-AT or IG-AT allocation. Figure 1 shows performance on FOGQ, MoCA, DGI, forward gait, FSST, and the FAB.

### Patients’ perspective

Participants’ impressions of AT as per the exit questionnaire were overall positive; participants strongly agreed (i.e., rating of “1”) that they enjoyed the intervention and would continue participating if possible. Four out of six participants expressed that the intervention encouraged them to be more physically active and helped them improve their balance, walking, and endurance. All participants, except EG1, agreed that the intervention encouraged them to be more mentally active. Participants appreciated the social aspects of AT, including interaction with other participants, class instructors, and volunteers.

## Discussion

Like AT, PT for people with PD incorporates visual or auditory cueing, which decreases the length and severity of FOG episodes. AT may be a unique program that positively impacts individuals with PD with FOG while providing PD-symptom relief. This study demonstrates that AT is appropriate and safe for those with FOG and that they could dance the leader or follower role. These results suggest that AT could reduce FOG. AT programs for people with PD-FOG could be applied and further adapted to meet the physical and non-physical needs of people with PD-FOG and engage partners and caregivers. Although result maintenance varied greatly at the 1-month post-test, positive changes from pre-test to post-test suggest that AT could be clinically relevant for treating individuals with PD-FOG, with marginal findings suggesting greater benefit for those who complete EG-AT training. Because PD is a progressive neurodegenerative disorder and FOG is a harbinger of worse outcomes, we interpret these results while considering an expected decline in performance over time, even with the best of pharmacological treatments. As such, maintaining scores post-intervention may be a positive effect of AT.

The improvements noted in the MDS-UPDRS parts I–IV and total score for several participants (regardless of group allocation) exceeded the minimal clinically important difference (MCID), indicating that these participants may have experienced considerable functional gains after the program (Mishra et al., 2024). Several participants were able to exceed the cutoff of 18 points on the DGI for safe ambulation, while three participants exceeded the threshold for fall risk on the FAB. Four out of six participants met or exceeded the MCID for the PDQ-39SI, suggesting improved health-related QOL in this highly vulnerable group of individuals with PD (Horváth et al., 2017). While these results are encouraging, the variability apparent in these scores underlines the struggles both clinicians and patients must face while treating FOG.

This case series provides an initial investigation of PD-FOG individuals’ motor, psychosocial, and cognitive responses to AT and for managing their safety, functional independence, and wellbeing. Future studies into the effect of AT on PD-FOG should include larger samples, longer follow-ups, and fine-tuning of AT by emphasizing more individualized FOG-related elements.

### Limitations

Participants did not dance in structured adapted tango classes in the month following treatment cessation. Participants ceased their structured dance classes but otherwise continued their daily activities. We examined participants after a washout largely to observe any maintenance of gains. At 1-month post-training, we observed maintenance, improvement, and declines with respect to baseline. In some cases, there were declines at the post-test that were followed by improvement at the 1-month post-test. Whether these improvements can be attributed to the dance program is not entirely clear. People with PD-FOG regularly experience fluctuations in their medication regimen and in their functional status that are not entirely explained by pharmacology, neuropathophysiology, or patient emotional status. Because of day-to-day fluctuations, possibly the participant had a low-performance day that was followed by a high functional status day at the 1-month post-test. Alternatively, it is possible that some improvements emerge some weeks after the training has ceased because participants had time to process and practice what they learned during their training program, resulting in enhanced performance at 1-month post-test. Further study is required to understand the variance in performance over time in people with PD-FOG. The case series model has low internal validity due to its inability to report a causal effect and its vulnerability to selection bias. Isolating IG and EG training presents difficulties due to some overlap in training between the two strategies. For example, EG-AT participants would sometimes anticipate instead of responding to their leader and initiate self-movement. IG-AT participants may have experienced EG cueing with music during the AT sessions. These overlaps should be further addressed in future studies.

## Data availability statement

The original contributions presented in the study are included in the article/Supplementary material, further inquiries can be directed to the corresponding author.

## Ethics statement

The studies involving humans were approved by the Institutional Review Board at Emory University School of Medicine. The studies were conducted in accordance with the local legislation and institutional requirements. The participants provided their written informed consent to participate in this study. Written informed consent was obtained from the individual(s) for the publication of any potentially identifiable images or data included in this article.

## Author contributions

AA: Conceptualization, Visualization, Formal Analysis, Writing – review & editing, Writing – original draft. AH: Project administration, Data curation, Writing – original draft, Writing – review & editing. AB: Project administration, Data curation, Writing – original draft, Writing – review & editing. SP: Formal Analysis, Visualization, Writing – original draft, Writing – review & editing. SW: Investigation, Writing – original draft, Writing – review & editing. KS: Writing – original draft. MC: Writing – original draft, Project administration. JP: Writing – original draft. AB: Writing – original draft. MT: Writing – original draft. CA: Writing – original draft. TP: Writing – review & editing. MH: Funding acquisition, Investigation, Methodology, Data curation, Conceptualization. Writing – original draft, Writing – review & editing.
