# Supplementary material for: Comparison of externally and internally guided dance movement to address mobility, cognition, and psychosocial function in people with Parkinson’s disease and freezing of gait: a case series
Source: Front Aging Neurosci. 2024 May 15;16:1372894. doi: 10.3389/fnagi.2024.1372894 (PMC11135342; doi:10.3389/fnagi.2024.1372894)
Supplement: Supplementary file 1 [file Presentation_1.pptx]

## Slide 1
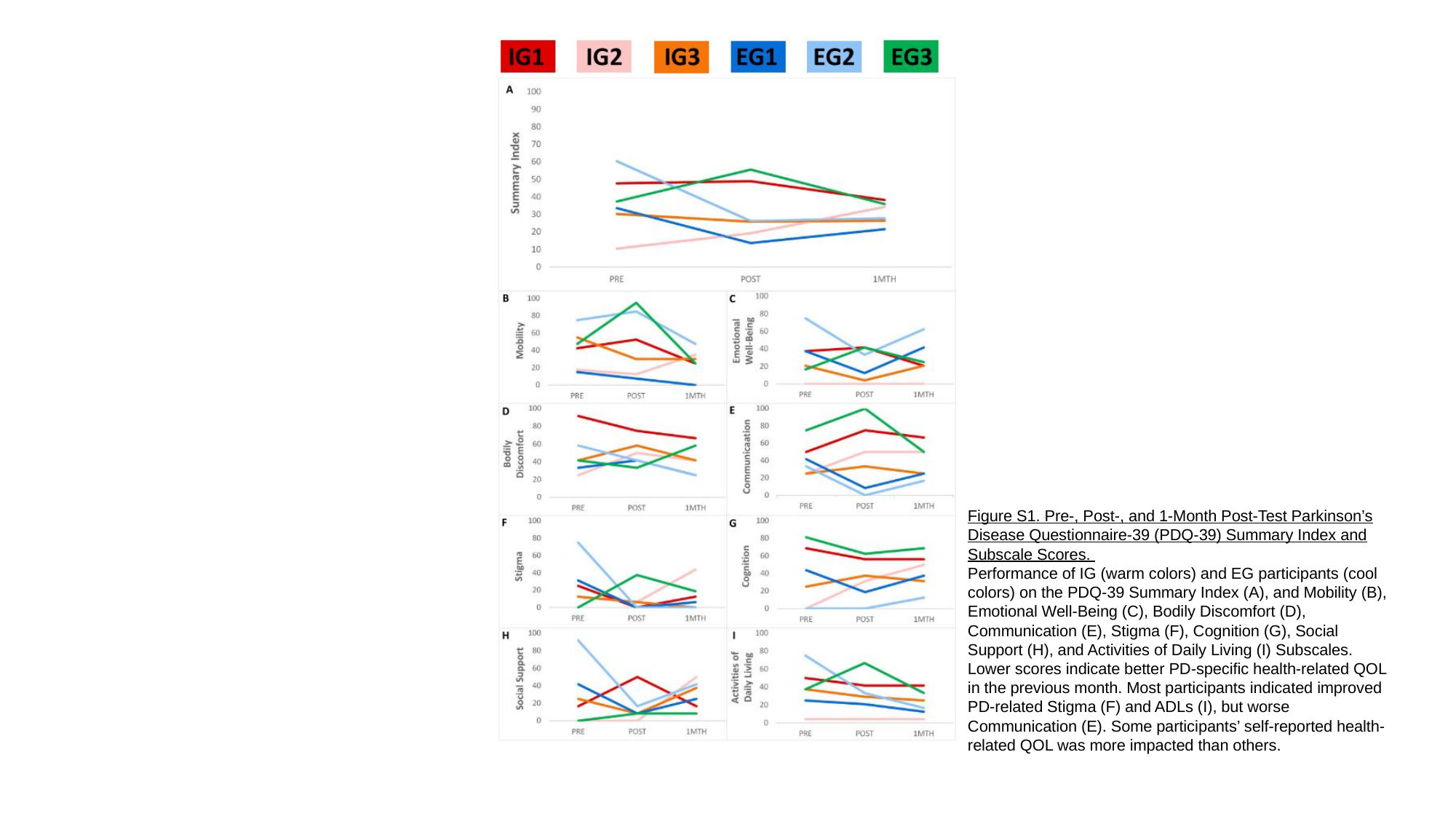

Figure S1. Pre-, Post-, and 1-Month Post-Test Parkinson’s Disease Questionnaire-39 (PDQ-39) Summary Index and Subscale Scores.
Performance of IG (warm colors) and EG participants (cool colors) on the PDQ-39 Summary Index (A), and Mobility (B), Emotional Well-Being (C), Bodily Discomfort (D), Communication (E), Stigma (F), Cognition (G), Social Support (H), and Activities of Daily Living (I) Subscales. Lower scores indicate better PD-specific health-related QOL in the previous month. Most participants indicated improved PD-related Stigma (F) and ADLs (I), but worse Communication (E). Some participants’ self-reported health-related QOL was more impacted than others.
