# Supplementary material for: Comparison of externally and internally guided dance movement to address mobility, cognition, and psychosocial function in people with Parkinson’s disease and freezing of gait: a case series
Source: Front Aging Neurosci. 2024 May 15;16:1372894. doi: 10.3389/fnagi.2024.1372894 (PMC11135342; doi:10.3389/fnagi.2024.1372894)
Supplement: Supplementary file 2 [file Data_Sheet_1.docx]

**Appendix 1: Participants’ Medical History**

**IG1**: A male who reported arthritis, gastric reflux, and a recent cataract surgery. He experienced FOG about once a day, lasting 11-30 seconds, without resulting in falls.

**IG2**: A female who reported breast cancer (in remission), hypertension, vertigo, hyperthyroidism, hypothyroidism, and glaucoma. She experienced FOG about once a day, with start hesitation, turning hesitation, and tight quarters hesitation episodes that last 1-2 seconds. She fell three times in the 6 months before the intervention. She had mild sleep and moderate urinary problems and had needed emergency medical care twice in the previous three years for her comorbidities.

**IG3**: A male who had been recently diagnosed with cognitive impairment and had received emergency medical care for blood clots in the previous three years. His FOG occurred once a week on average for approximately 1-2 seconds, with festinating gait about once a day. He fell three times in the 6 months before the intervention and reported that his falls are due to FOG episodes. Sleep issues, fatigue, and pain impacted him daily.

**EG1**: A male with no comorbid medical conditions. He had sought emergency medical care in the previous three years to obtain sutures after a fall. His FOG was daily, lasting between 1-30 seconds and he had experienced six falls in the 6 months before the intervention. He described symptoms of mild levels of depression and anxiety, moderate difficulty with sleeping, urinary incontinence, saliva and drooling, mild tremor, and mild difficulty with eating.

**EG2**: A female who reported hypertension, heart problems, depression, and arthritis. She experienced FOG several times daily and her typical start hesitation lasted >10 seconds. She also experienced hesitation in turning, destination, and tight quarter hesitation lasting 11-30 seconds. She fell seven times in the 6 months before the intervention.

**EG3**: A female, the youngest participant, who had had PD the longest, reported no comorbid medical conditions but had freezing episodes often throughout the day that sometimes lasted ≥30 seconds. She reported daily falls (182) within the 6 months before the intervention. She had the most observable freezing and wore kneepads habitually.
